# Characterization of Salivary Secreted Proteins That Induce Cell Death From *Riptortus pedestris* (Fabricius) and Their Roles in Insect-Plant Interactions

**DOI:** 10.3389/fpls.2022.912603

**Published:** 2022-07-04

**Authors:** Yumei Dong, Xingge Huang, Yuxia Yang, Jifen Li, Meiqian Zhang, Hui Shen, Yanrong Ren, Xinyu Li, Jiale Tian, Danyu Shen, Daolong Dou, Ai Xia

**Affiliations:** College of Plant Protection, Nanjing Agricultural University, Nanjing, China

**Keywords:** *Riptortus pedestris*, salivary gland, cell death-inducing proteins, HAMP, staygreen

## Abstract

*Riptortus pedestris* (Fabricius) is a polyphagous hemipteran crop pest that mainly feeds on the leguminous plants, resulting in shriveled and dimpled seeds. With recent several outbreaks in the Huang-Huai-Hai region of China, as well as in South Korea and Japan, this species has caused enormous economic losses to soybean crops. In the present study, we found that *R. pedestris* feeding results in local lesions at the infestation sites. To identify the key effectors that induce plant damage during feeding, the salivary glands of *R. pedestris* were dissected for transcriptome sequencing, and 200 putative secreted proteins were transiently expressed in *N. benthamiana*. Among them, three intracellular effectors (RP191, RP246, and RP302) and one apoplastic effector (RP309) were identified as necrosis-inducing proteins (NIPs), which also triggered the reactive oxidative burst. Yeast signal sequence trap and qRT-PCR analysis suggested that these proteins might be secreted into plant tissue during *R. pedestris* infestation. Pathogenicity assays revealed that RP191, 246, and 302 promote *Phytophthora capsici* infection or induce *Spodoptera litura* feeding by inhibiting plant immunity. RP302 is localized to the cytoplasm and nuclei, while RP191 and 246 are endoplasmic reticulum (ER) resident proteins. RP309 stimulates the expression of PTI marker genes, and its induced cell death depends on co-receptors NbBAK1 and NbSOBIR1, indicating that it is a HAMP. Bioinformatics analysis demonstrated that four NIPs are recently evolved effectors and only conserved in the Pentatomidae. In this study, saliva-secreted proteins were used as the starting point to preliminarily analyze the harm mechanism of *R. pedestris*, which might provide a new idea and theoretical basis for this species control.

## Introduction

*Riptortus pedestris* (Fabricius) of the family Alydidae, order Hemiptera, is a polyphagous agricultural pest whose hosts include various leguminous plants, fruit trees, rice, flowering plants, and medical plants (Mainali et al., 2014), but prefer to feed on leguminous crops such as soybeans (*Glycine max*), cowpeas (*Vigna unguiculata*), and white kidney beans (*Phaseolus vulgaris*) (Lim and Mainali, 2013). *R. pedestris* is widely spread in East Asia, including South Korea, Japan, India, and China, and has been regarded as a severe crop pest since 2000. In South Korea, *R. pedestris* resulted in up to 70% of economic losses in soybean fields (Kim and Lim, 2010). The peak population of *R. pedestris* migrates into the fields during the seed maturity stage from pod filling to harvest, causing dimpled and shriveled seeds (Mainali and Lim, 2012). In China, the rate of *R. pedestris* infestations has recently increased sharply (Li et al., 2021). Damages caused by this pest are typically characterized as “staygreen syndrome” or “*zhengqing*,” due to the lack of leaf senescence and increased pod abortion rates. Staygreen syndrome is most prevalent in the Huang-Huai-Hai river basin, including Henan, Beijing, Shaanxi, and some parts of the Shandong provinces, resulting in tremendous yield losses for soybean growers (Li et al., 2019). Besides the direct feeding damage, *R. pedestris* also serves as a vector to transmit plant diseases such as *Eremothecium coryli* and *Eremothecium ashbyi* (Kimura et al., 2008).

Salivary proteins play an important role in the co-evolution of plants and herbivorous insects, which contain various enzymes, proteins, and compounds (Jiang et al., 2019; Nalam et al., 2019). During the feeding process, herbivorous insects inject these components into plant tissue which are thought to be potential effectors to aid in feeding (Huang H. J. et al., 2019; Dong et al., 2020). But Some herbivore-derived small molecules or proteins which are known as herbivore-associated molecular patterns (HAMPs), are recognized by plant pattern recognition receptors (PRRs) on the cytoplasm membrane to trigger plant “pattern-triggered immunity” (PTI). However, few types of HAMP are reported, and most mechanisms are unclear. Most HAMPs can be characterized as fatty acid derivatives, enzymes, and other proteins (Snoeck et al., 2022). The brown planthopper, *Nilaparvata lugens* secrets mucin-like protein (NlMLP) elicits plant jasmonic acid (JA) signaling pathway (Shangguan et al., 2018). Very recently, using forward-genetic mapping, the PRR of a caterpillar elicitor inceptin, termed inceptin receptor (INR), was identified from a legume species (Steinbrenner et al., 2020).

Other types of effectors, including some apoplastic effectors and intracellular effectors, utilize a variety of tactics to suppress plant defense responses to establish successful feeding (Stahl et al., 2018). For instance, Aphid macrophage migration inhibitory factor (MIF) inhibits major plant immune responses, such as the expression of defense-related genes, callose deposition, and hypersensitive cell death (Naessens et al., 2015). *Acyrthosiphon pisum* Armet, *Bemisia tabaci* Bt56, and *Helicoverpa armigera* HARP1 interact with the JA pathway (Chen et al., 2019; Cui et al., 2019; Xu et al., 2019). *Apolygus lucorum* and *B. tabaci* salivary proteins Al6 and Btfer1 inhibit reactive oxygen species (ROS) (Su et al., 2019; Dong et al., 2020), and *B. tabaci* Bsp9 inhibits MAPK to promote insect piercing (Wang et al., 2019). The small brown planthopper (SBPH) *Laodelphax striatellus* calcium-binding protein (LsECP1) acts as an effector to impair host rice defense responses and promotes SBPH performance (Tian et al., 2021). However, there is limited information available on *R. pedestris* HAMPs or effectors.

Given the devastating economic losses caused by *R. pedestris* annually and the lack of salivary effectors for this species, the aims of the present study were (1) to identify the candidate effectors secreted by *R. pedestris* during feeding that contribute to plant cell death, and (2) to explore the molecular mechanisms by which these effectors manipulate plant immunity and facilitate feeding. Our findings could contribute to the improved management strategies that will alleviate the economic burden caused by this insect. In this study, 418 salivary gland highly expressed secretory proteins were identified by salivary gland transcriptome analysis, and 200 of them were functionally screened. We identified three intracellular effectors that inhibited plant immunity and one intercellular elicitor that stimulated plant immunity.

## Materials and Methods

### Insect and Plants Materials

*Riptortus pedestris* collected from soybean fields in Shandong and Nanjing, China in 2019, has been reared in the insectary room for more than 2 years. Nymphs and adults were reared in cages (40 cm × 40 cm × 40 cm) at 25°C under a 16L:8D h photoperiod and fed with potted soybean plants or dried seeds. *Nicotiana benthamiana* and soybean (cv. Williams) was cultivated at 25°C and 60% of relative humidity under a 16:8 h (light:dark) photoperiod. *Spodoptera litura* was kept at 25 ± 1°C with a 16:8 h (light:dark) photoperiod, and larvae were reared on an artificial diet made from wheat germ and soybean powder.

### Salivary Gland Dissection and RNA Extraction

The salivary glands were dissected gently under a stereomicroscope by slowly removing the head and other tissue, such as fat body droplets, and photographs were acquired with a TOUPCAM digital camera before immediately transferring the glands to liquid nitrogen. Total RNA was extracted from 10 whole bodies of *R. pedestris* or 100 salivary glands using the RNA simple Total RNA Kit according to the manufacturer’s protocol (Tiangen, China).

### Bioinformatics Analysis

Two libraries were constructed using RNA extracted from the dissected salivary glands and the whole body of *R. pedestris*. The two libraries were subjected to the Illumina HiSeq X Ten system to 150-bases paired-end reads. Read trimming was performed using Trimmomatic to remove adapter sequences and low-quality base calls (Bolger et al., 2014). The trimmed reads were used to perform *de novo* assembly using Trinity assembler with default parameters (Grabherr et al., 2011). The potential protein coding genes were predicted from the assembled transcripts using TransDecoder with the minimum length of 50 amino acids. The signal peptide of each protein was predicted using SignalP v3.0 with HMM probability ≥ 0.9 (Bendtsen et al., 2004). Transmembrane helices in proteins were predicted using TMHMM Server v2.0 (Krogh et al., 2001). Potential domains in proteins were predicted using the Pfam database (EMBL-EBI). To identify homologs of candidate effectors, a Blastp search was performed against the non-redundant NCBI protein sequence database with an E threshold of 1e-5 (NCBI).

### Cloning of Candidate Effectors and Plasmid Construction

Candidate effectors were amplified from *R. pedestris*. For overexpression in *N. benthamiana*, the candidate effectors were cloned into plant expression vector pBinGFP2 and pBin3HA [a plasmid vector carrying green fluorescent protein (GFP) or HA tag] using a ClonExpress II One Step Cloning Kit (Vazyme, Nanjing, China).

### *Agrobacterium tumefaciens* Infiltration Assays

The positive constructs were transferred into *Agrobacterium tumefaciens* strain GV3101 by electroporation. Recombinant strains of *A. tumefaciens* were cultured in Luria-Bertani (LB) medium at 28°C with shaking at 200 rpm for 36 h. The cells were then washed and re-suspended in infiltration buffer (10 mM MgCl_2_, 10 mM MES pH = 5.7, 150 μM acetosyringone) to an optical density (OD) of 0.4–0.6 at 600 nm. The suspensions were infiltrated into *N. benthamiana* leaves using a syringe.

### Oxidative Burst Assay

Reactive oxygen species levels were tested by DAB (3, 3′-diaminobenzidine) staining. The candidate effectors were expressed in *N. benthamiana* leaves for 2 days, and immersed in 1 mg/ml DAB solution (Sigma-Aldrich, St. Louis, CA, United States) at 25°C for 6 h, then decolorized in industrial alcohol and preserved in 30% glycerol. Finally, photographs were taken at natural light.

### Electrolyte Leakage Assay

Cell death was evaluated by measuring the amount of ion leakage in leaves (Ai et al., 2021; Yang et al., 2021). Five 9 mm-leaf disks were washed three times with distilled water and then soaked in 5 mL of distilled water for 3 h at room temperature. The conductivity of the soaking solution (A) was measured using a conductivity meter (Con 700; Consort, Turnhout, Belgium). Then the suspension was moved to a sealed pipe in a water bath at 95°C for 25 min, cooled to room temperature, and the conductivity B was measured. The ion leakage value was calculated as A/B × 100%.

### Western Blots

After the proteins were expressed in *N. benthamiana* leaves for 36–48 h, the leaves were collected to extract total protein using a previously described method (Si et al., 2021). The isolated proteins were separated on a 10% SDS-PAGE, blotted and incubated with mouse anti-GFP monoclonal antibodies (1:5,000; Cat. No. M20004, Abmart, Shanghai, China) and anti-HA monoclonal antibodies (1:5,000; Cat. No. M20003, Abmart, Shanghai, China). Then the membrane was incubated with goat anti-mouse IRDye 800CW (Cat. No. 926-32210, Odyssey, Li-Cor). And finally washed and visualized using Odyssey with excitation at 700 and 800 nm.

### Confocal Microscopy

To demonstrate the localization of candidate effectors, patches of agro-infiltrated *N. benthamiana* leaves were cut when expression was at its maximum at 36 h and analyzed using an LSM 710 confocal microscope under 20× and 60× objective lenses (Carl Zeiss, Jena, Germany).

### Quantitative Real-Time Polymerase Chain Reaction Analysis

*Riptortus pedestris* cDNA was synthesized from total RNA using the HiScript II Q RT SuperMix for qPCR, and real-time PCR was performed using SYBR Green Master Mix (Vazyme, Nanjing, China). Quantitative real−time polymerase chain reaction (qRT-PCR) was performed on an ABI PRISM 7500 real-time PCR system (Applied Biosystems, Waltham, CA, United States) under the following conditions: 95°C for 5 min, 95°C for 10 s, and 60°C for 34 s for 40 cycles to calculate the cycle threshold values, followed by a melting program of 95°C for 15 s, 60°C for 1 min, and 95°C for 15 s to obtain melt curves. The relative expression levels of genes were normalized to β-actin as the reference gene. At least three biologically independent replicates were carried out for each sample.

### Yeast Signal Sequence Trap System

The yeast signal sequence trap system assay was performed using a previously described method (Bottger et al., 2006; Ai et al., 2021). Briefly, PSUC2-derived plasmids were transformed into yeast, which was deposited on complete minimal plates lacking tryptophan (CMD-W) plates (0.67% Yeast nitrogen base without amino acids, 0.074% W dropout supplement, 2% Sucrose, 0.1% Glucose, and 2% Agar). The positive clones were then transferred to YPRAA plates (1% Yeast extract, 2% Peptone, 2% Raffinose, and 2 μg/mL Antimycin A) to detect invertase secretion. Invertase activity was monitored by measuring 2, 3, 5-triphenyltetrazolium chloride (TTC) reduction to the insoluble red triphenylformazan.

### Pathogen Infection and Insect Feeding Assays

The *Phytophthora capsici* strain LT263 was cultured at 25°C in the dark on 10% (v/v) V8 juice medium. To assay *P. capsici* infection, *N. benthamiana* leaves transiently expressing candidate effectors or control proteins (GFP) were grown in a greenhouse for 24 h, and infection assays were conducted using a previously published method (Zhou et al., 2021). The lesion areas (cm^2^) were measured after 36 or 48 h incubation at 25°C using ImageJ software. The insect feeding assay was performed according to a previously published method (Dong et al., 2020). The blade damage area was measured after 24 h incubation at 25°C using ImageJ software. These assays were repeated at least three times. Significant differences were calculated using a Student’s *t*-test.

### Virus-Induced Gene Silencing in *Nicotiana benthamiana*

pTRV1, pTRV2: BAK1, pTRV2: SOBIR1, pTRV2: GFP, and pTRV2: PDS plasmid constructs were transformed into *A. tumefaciens* GV3101, and then, Agrobacterium strains harboring the vectors above were first mixed with the strain harboring pTRV1 vector with a 1:1 ratio in the infiltration solution (10 mM MgCl2, 500 mM MES pH 5.7, and 150 μM acetosyringone) to a final OD600 of 0.6.

The effectiveness of the virus−induced gene silencing (VIGS) assay was assessed according to the phenotype of PDS, as described previously. The silencing efficiency of NbBAK1 and NbSOBIR1 were determined by qRT-PCR. The NbEF1a was used as an endogenous control. The experiments were repeated three times. Primers used in this study are listed in Supplementary Table 1.

## Results

### *Riptortus pedestris* Feeding Induces Soybean Plant Cell Death

**FIGURE 1:**
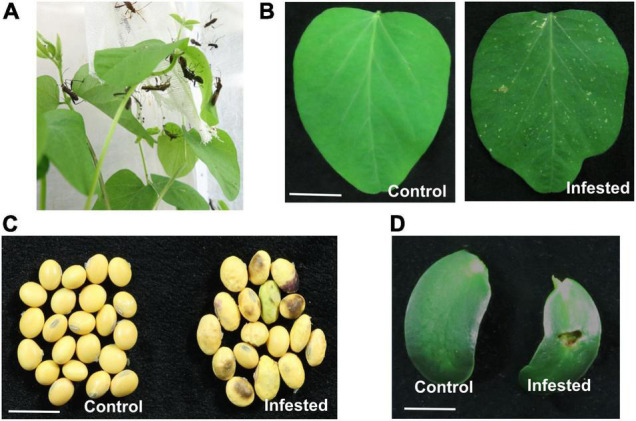
*Riptortus pedestris* feeding induces soybean cell death. **(A)** *Riptortus pedestris* feeds on soybean leaves. **(B–D)** *Riptortus pedestris* induces cell death on soybean leaves, seeds, and cotyledons. Infested leaves were photographed 5 days after adult feeding. Control leaves means were not fed upon. Bar = 1 cm.

In the field, the damage caused by *R. pedestris* manifests as “staygreen syndrome” and aborted seeds (Li et al., 2019). Field cage assessment also revealed that both nymphs and adults of *R. pedestris* can feed on the leaves, stems, flowers, pods, and seeds (Li et al., 2021), but authors failed to provide detail description of damage symptoms of *R. pedestris* on plants. In the present study, both nymphs and adults of *R. pedestris* fed on the deferent developmental stages of soybean plants, including the leaves, cotyledon, and seeds. *R. pedestris* inserted their needle-liked mouthparts into the front or back sides of leaflets to obtain nutrients and water (Figure 1A). This feeding led to multiple pinhole-shaped dots and few whitish streaks at the feeding sites on soybean leaves (Figure 1B). After feeding on soybean pods for 2 weeks, the seeds were shriveled and shrunken in comparison with round and fully developed normal seeds, and the seed color was brown and dark, indicating that infested seeds develop abnormally with severe tissue necrosis (Figure 1C). Damage at the infestation sites of cotyledons manifested as a large hole and local necrosis (Figure 1D).

### Sequencing of *Riptortus pedestris* Salivary Gland Transcriptome and Prediction of Candidate Effectors

**FIGURE 2:**
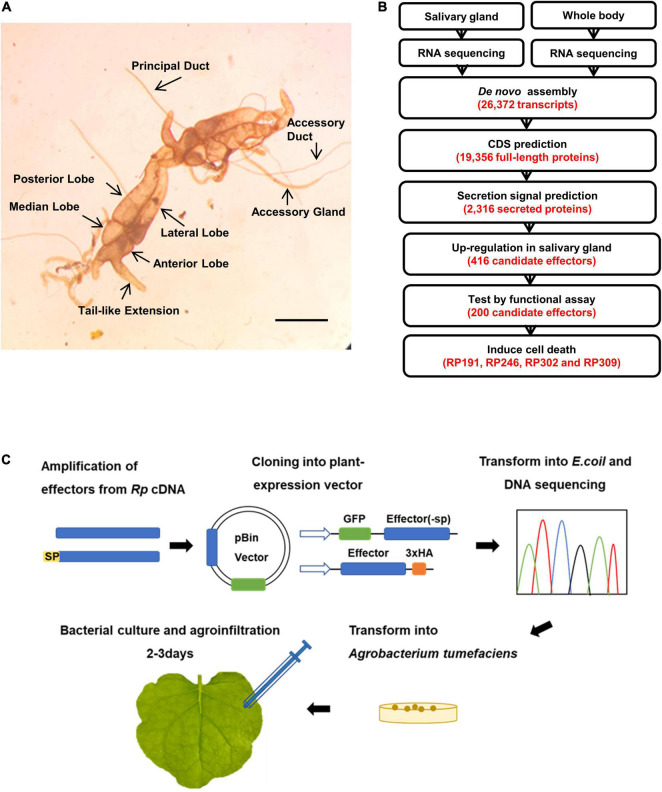
Sequencing of *R. pedestris* salary gland transcriptome and prediction of candidate effectors. **(A)** The salivary gland structure of *R. pedestris*. Bar = 5 mm. **(B)** Bioinformatics pipeline for the identification of candidate effectors from *R. pedestris* salivary gland. **(C)** Flow chart of functional analyses of candidate effectors. *Rp, R. pedestris*.

To identify secreted protein factors of *R. pedestris* that induce plant cell death, the salivary glands were dissected from adult *R. pedestris*. Each *R. pedestris* has a pair of salivary glands, one on the left and one on the right (Figure 2A). Since no previous report had defined the salivary glands of *R pedestris*, we defined them as four major lobes: the anterior, lateral, median, and posterior lobes, and two ducts: the principal and accessory ducts (Figure 2A). The morphology is very similar to the salivary glands of *Riptortus dentipes* (Soyelu et al., 2007).

**FIGURE 3:**
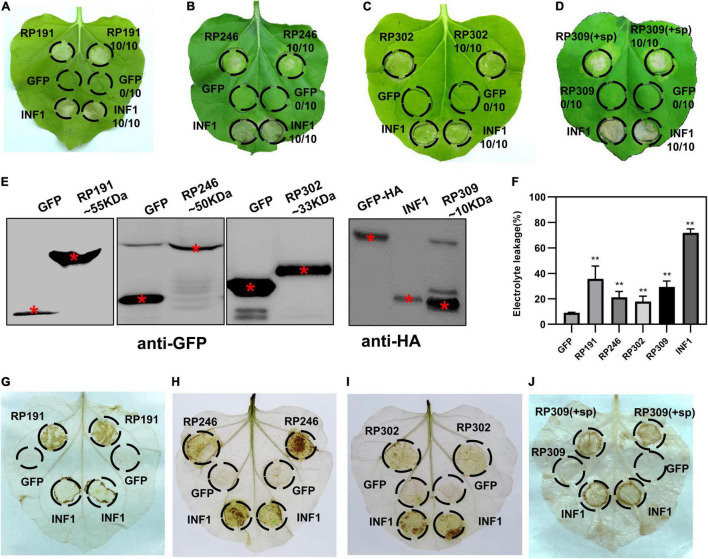
Identification of necrosis-inducing proteins (NIPs) in *Nicotiana benthamiana*. **(A–D)** Necrosis inducing proteins (NIPs) induce cell death in *N. benthamiana* leaves. Green fluorescent protein (GFP) was used as the negative control and INF1 was the positive control. GFP, GFP-NIPs, and INF1 were expressed in *N. benthamiana* leaves *via* agroinfiltration. The leaves were photographed at 4 days after agroinfiltration. All experiments were repeated 10 or more times. **(E)** Immunoblot analysis of GFP, NIPs, and INF1 expressed in *N. benthamiana*. Red asterisks indicated the right protein bands. **(F)** Quantification of electrolyte leakage in *N. benthamiana* leaves expressing NIPs. All the experiments were replicated three times and then analyzed using Student’s *t*-test (**P < 0.01). **(G–J)** ROS accumulation associated with each NIP. DAB staining was performed at 2 days after infiltration on leaves containing GFP, INF1, and NIPs.

The total RNA obtained from dissected salivary glands and the whole body were subjected to RNA-Seq sequencing, yielding a total of 113.45 million paired-end reads. The adaptors and poor-quality reads were removed by data trimming. The quality-trimmed reads were used for *de novo* transcriptome assembly using Trinity. The final assembly consisted of 26,372 transcripts. These transcripts had an average length of 1059 bp and N50 value of 1529 bp. A total of 19,356 ORFs were identified among Trinity transcript sequences using TransDecoder (Figure 2B). Considering that effectors usually contain the classical signal peptides, the predicted CDS proteins were analyzed using SignalP v3.0 and TMHMM v2.0, and 2,316 proteins with an N-terminal signal peptide and without transmembrane helices were identified. We assumed that *R. pedestris* effectors would be highly expressed in the salivary glands, and 418 proteins were identified as upregulated more than twofolds in the salivary glands compared with in the whole body (Figures 2B, 3). Thus, 418 *R. pedestris* secreted proteins were considered as candidate salivary gland effectors. Further analysis showed that the average length of the 418 proteins was 152 aa, and 47% of proteins were cysteine-rich secreted proteins (cysteine content of 3% or more). Annotation against NCBI non-redundant (nr) protein database using Blast showed that over 57% of the 418 proteins didn’t share similarity with nr protein sequences.

### Identification of Necrosis-Inducing Proteins in *Nicotiana benthamiana*

The efficient model plant transient expression system *N. benthamiana* was used to screen potential salivary gland proteins of *R. pedestris*. A total of 200 candidate effectors with high upregulated expression were selected from 418 secreted proteins. And we cloned the full-length sequences of 200 candidate effectors and de-signaling peptide sequences from *R. pedestris* CDNA and then constructed them into corresponding plant expression vectors to express in *N. benthamiana* (Figure 2C). An empty vector (GFP) was used as the negative control, and *Phytophthora infestans* PAMP INF1 was used as the positive control. Among the 200 candidate salivary gland proteins, RP191, RP246, and RP302 are able to induce cell death in intracellular. And full-length RP309 (RP309 + sp) but not RP309 (without signal peptide) triggered cell death. So RP309 triggered cell death in *N. benthamiana* when secreted to the apoplast. These four proteins subsequently referred to as necrosis-inducing proteins (NIPs) (Figures 3A–D). Western blot analysis showed that all recombinant proteins, GFP, GFP-NIPs, and INF1-HA, were expressed correctly in *N. benthamiana* (Figure 3E).

To quantify the degree of cell death triggered by the four salivary gland NIPs, cell membrane stability was detected by measuring the amount of ion leakage in *N. benthamiana* leaves, revealing that the four NIPs caused significantly elevated electrolyte leakage compared with GFP-expressing infiltrated leaves (Figure 3F). The ROS accumulation was also measured with DAB staining. A strong oxidative burst was observed in *N. benthamiana* leaves expressing RP191, RP246, RP302, and RP309 proteins (Figures 3G–J). Taken together, these results suggest that *R. pedestris* salivary gland proteins RP191, RP246, RP302, and RP309 can trigger cell death and ROS accumulation in *N. benthamiana*, meanwhile RP309 was a potential HAMP.

### Necrosis-Inducing Proteins Are Secreted Proteins and Are Highly Expressed in Salivary Glands

**FIGURE 4:**
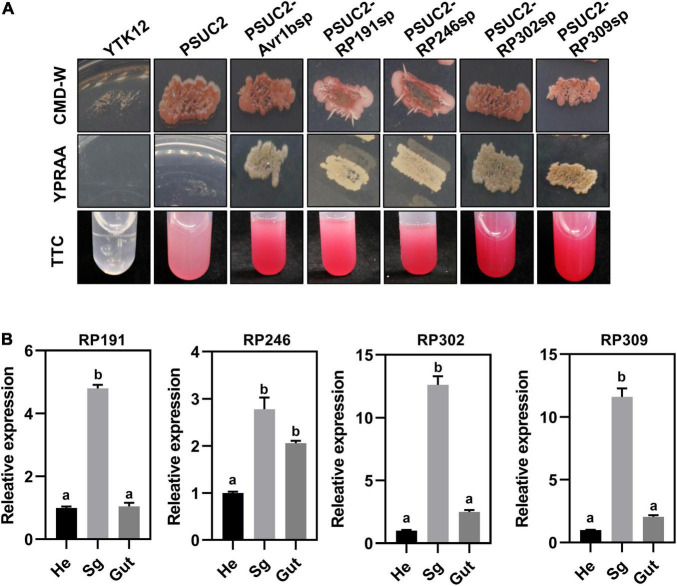
Necrosis-inducing proteins (NIPs) are secreted proteins and are highly expressed in the salivary glands. **(A)** Detection of N-terminal signal peptide (SP) of NIPs by yeast secretion system. The yeast YTK12 strain carrying PSUC2-Avr1b and NIP signal peptides could grow on CMD-W and YPRAA media, and reacted with TTC to form a red product, indicating the secretion of invertase. YTK12 stain and YTK12 carrying the pSUC2 vectors were the negative controls. **(B)** Transcription expression patterns of NIPs in different tissue (Sg, salivary gland; Hd, head; Gut, gut). Data were normalized to β-actin gene expression. Different letters above the bars indicate significant differences.

All *R. pedestris* salivary gland NIPs were predicted to contain a signal peptide according to the SignalP 5.0 HMM algorithm. A yeast secretion assay was therefore performed to validate the secretion of the four signal peptides of RP191, RP246, RP302, and RP309 by constructing each predicted signal peptide into the pSUC2 plasmid and then transforming into an invertase drug-deficient yeast strain YTK12. The previously published signal peptide of *Phytophthora sojae* effector Avr1b was used as the positive control (Dou et al., 2008). The untransformed YTK12 strain and YTK12 with pSUC2 (empty vector) were used as negative controls. The resultant constructs, pSUC2-Avr1b-SP, pSUC2-RP191-SP, pSUC2-RP246-SP, pSUC2-RP302-SP, and pSUC2-RP309-SP, enabled the invertase deficient yeast to grow on YPRAA medium, and discolored the TTC solution, unlike the negative control YTK12 and pSUC2, indicating that the predicted signal peptides of the four NIPs are functional and these proteins are likely secreted from *R. pedestris* (Figure 4A).

To further verify that the four NIPs might be secreted from the salivary glands, the relative transcriptional levels of the NIPs were measured in different body parts of *R. pedestris* by dissecting the whole insect into the head, gut, and salivary glands. The relative expression of RP191, RP246, RP302, and RP309 was higher in the salivary gland than in the head and gut (Figure 4B). This suggests that these NIPs might be secreted from the salivary gland into plant cells during feeding.

### Necrosis-Inducing Proteins Affect Plant Pathogen Infection and Insect Feeding

**FIGURE 5:**
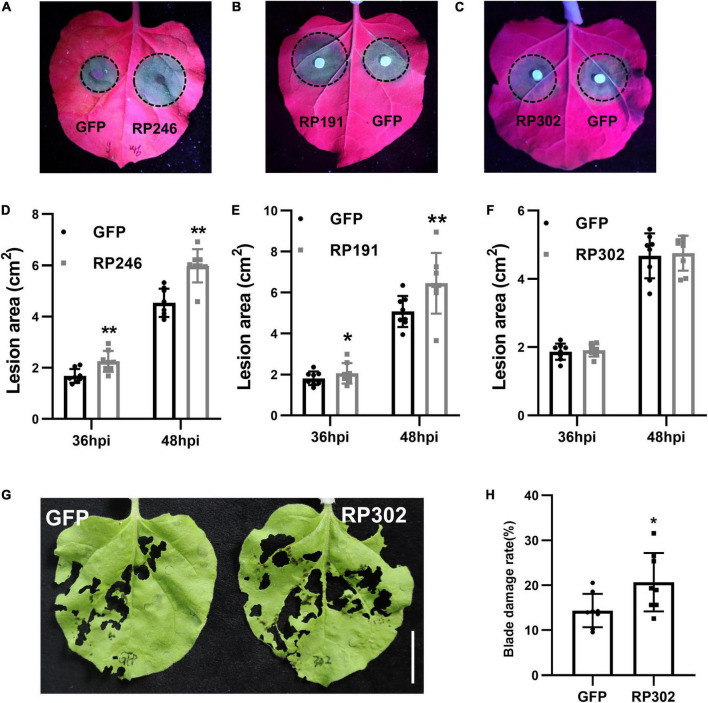
Necrosis-inducing proteins (NIPs) affect plant pathogen infection and insect feeding. **(A–C)** *Phytophthora capsici* infection assays with *N. benthamiana* leaves expressing green fluorescent protein (GFP) or GFP-NIPs. Representative photographs were taken under UV light at 48 hpi and the lesion area was measured at 36 and 48 hpi **(D–F)**. Average lesion areas were calculated from eight leaves. **(G,H)** *Spodoptera litura* feeding on leaves expressing GFP, GFP-RP302. Similar results were obtained in three independent experiments. Asterisks at the top of the bars indicate statistical significance. Error bars represent SD (**P < 0.01, *P < 0.05, Student’s *t*-test).

*Riptortus pedestris* salivary gland proteins RP191, RP246, and RP302 are candidate effectors that might alter host plant immunity. To explore the effect of these NIPs on plant resistance to pathogens, we transiently overexpressed GFP and GFP–NIPs in *N. benthamiana* for 24 h, and then inoculated with the hemibiotrophic oomycete pathogen *P. capsici*. At 36 and 48 h after inoculation, the pathogen infection areas were measured and statistically analyzed. The lesion sizes in tobacco leaves with RP246 and RP191 were significantly larger than in the leaves expressing GFP (Figures 5A,B,D,E). However, the lesion areas were not significantly different in leaves expressing GFP and RP302 (Figures 5C,F). This suggests that RP191 and RP246 may inhibit *N. benthamiana* immunity and promote *P. capsici* infection, while RP302 do not affect plant susceptibility to *P. capsici*.

Since *N. benthamiana* is a natural host of *S. litura*, we performed feeding assays with *S. litura* and tobacco leaves expressing RP302. RP302 induces *S. litura* to feed more on *N. benthamiana* leaves than the negative control expressing GFP, suggesting that RP302 facilitates *S. litura* feeding behavior (Figures 5G,H). These results demonstrate that RP191, RP302, and RP246 are potential effectors with the ability to block *N. benthamiana* immunity and facilitate plant pathogen colonization or insect feeding.

### The Cellular Localizations of Intracellular Necrosis-Inducing Proteins

**FIGURE 6:**
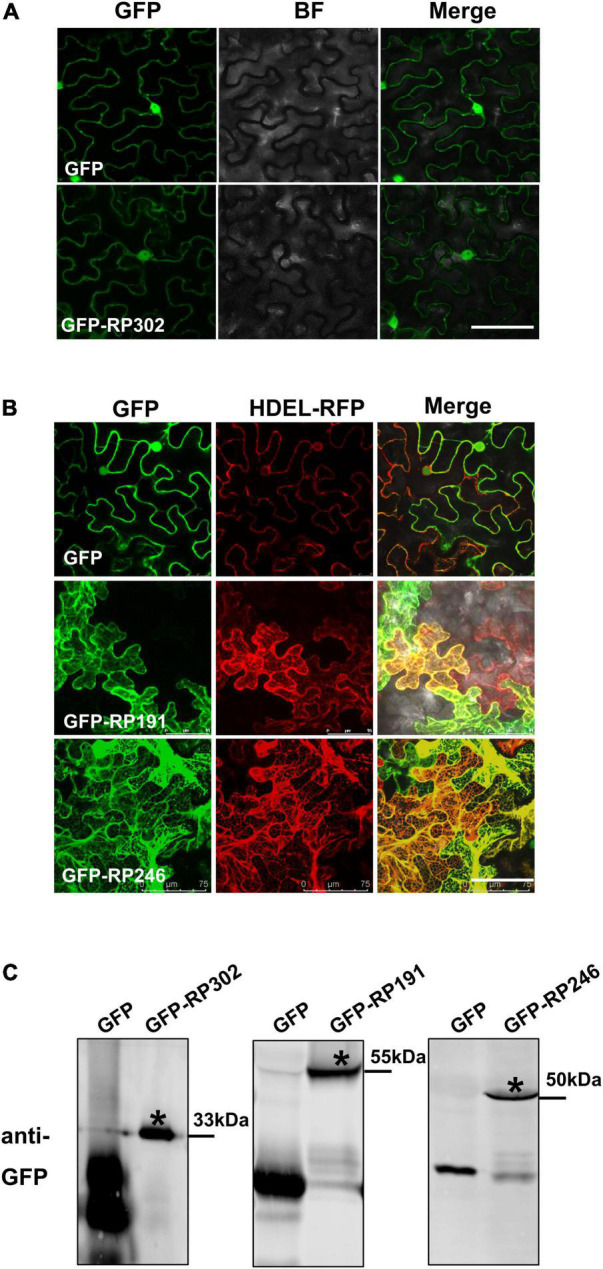
The cellular localization of intracellular NIPs. **(A)** The cellular localization of GFP-RP302. **(B)** The cellular localization of GFP-RP246 and GFP-RP191. Green and red fluorescent protein (GFP and RFP) fusion proteins were transiently expressed in *N. benthamiana* using agroinfiltration and localized at 36 h after infiltration. Bar = 75 μm. **(C)** Representative immunoblots showing the proteins levels of GFP, GFP-NIPs after transient expression in *N. benthamiana* leaves. Asterisk represents the destination strip.

The subcellular localization of effectors provides valuable information for deciphering their mode of action on plant cells. RP191, RP246, and RP302 proteins were transiently expressed in *N. benthamiana*, and were fused with GFP to visualize their cellular locations. The marker protein GFP was visible in both the cytoplasm and nucleus of tobacco cells (Figure 6A). RP302 were localized inside the nucleus and cytoplasm, similarly to the GFP control (Figure 6A). Our preliminary data suggests that RP191 and RP246 are likely localized to the endoplasmic reticulum (ER). To confirm this, GFP, GFP-RP191, GFP-RP246, and the mRFP-HDEL, a known ER retention signal, were expressed in *N. benthamiana* leaves. The green fluorescent signals of RP191 and RP246 were co-localized with the HDEL signal, while the GFP control was not (Figure 6B), suggesting that RP191 and RP246 are localized to the ER. Immunoblotting analysis confirmed that GFP-NIPs were expressed at sufficient levels in *N. benthamiana* leaves (Figure 6C).

### RP309 Activates the Defense Response in *Nicotiana benthamiana*

**FIGURE 7:**
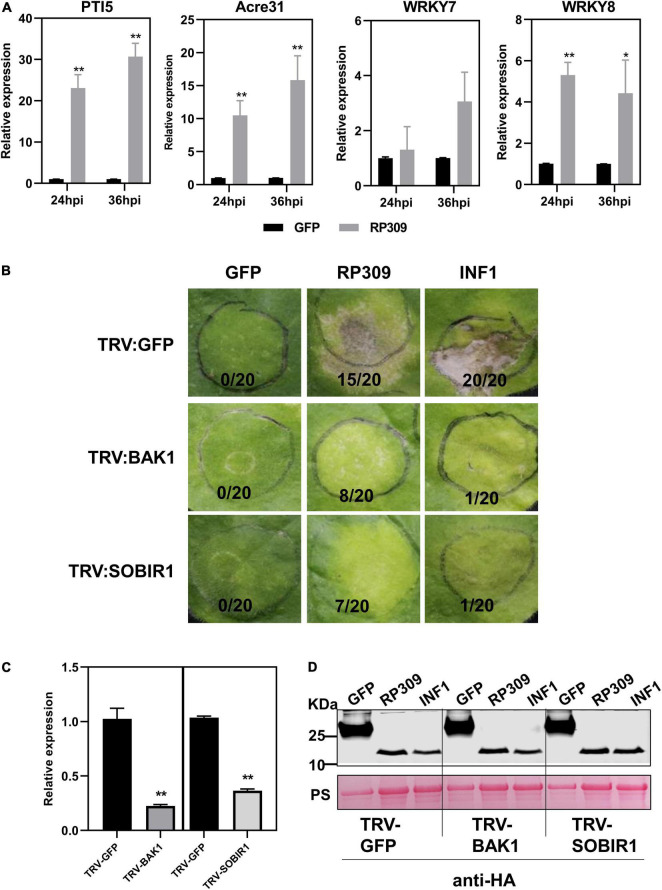
RP309 activates the defense response in *N. benthamiana*. **(A)** Relative expression of the PTI marker genes in *N. benthamiana* leaves expression GFP-HA or RP309-HA. Similar results were obtained in three independent experiments. **(B)** TRV–based virus–induced gene silencing (VIGS) vectors were used to silence NbBAK1 and NbSOBIR1. TRV–GFP was used as a negative control. Photographs were taken 5 days post-infection. **(C)** The expression levels of NbBAK1 and NbSOBIR1 after VIGS–mediated silencing, as determined by quantitative real–time polymerase chain reaction (qRT–PCR). Asterisks at the top of the bars indicate statistical significance. Error bars represent ± SD (Student’s *t*-test, **P < 0.01; *P < 0.05). **(D)** Immunoblot analysis of GFP, RP309, and INF1 expressed in *N. benthamiana* leaves. Asterisk represents the destination strip.

According to the previous results, we speculated that RP309 was a HAMP secreted by *R. pedestris*. To determine whether RP309 can induce defense responses in *N. benthamiana*, RP309 and GFP (negative control) were transiently expressed in *N. benthamiana* leaves. We then detected the relative expression of four PTI-marker genes PTI5, Acre31, WRKY7, and WRKY8 (Nie et al., 2019; Yang et al., 2022). The qRT-PCR assay showed that PTI5, Acre31, and WRKY8 were remarkably activated, but not WRKY7 (Figure 7A).

BAK1 and SOBIR1 are co-receptors of different PRRs, which can recognize PAMPs and HAMPs (Abdul Malik et al., 2020; Zhang et al., 2020). Our study suggests that RP309 induces cell death when secreted into the apoplast in *N. benthamiana*. However, it remains unclear whether RP309 is HAMP, and whether both NbBAK1 and NbSOBIR1 are involved in RP309-triggered cell death. We employed the VIGS system to silence NbBAK1 and NbSOBIR1 in *N. benthamiana* leaves. A previous study showed that the PAMP INF1, induced cell death in the apoplast of *N. benthamiana*, and this response required the PRRs NbBAK1 and NbSOBIR1 (Domazakis et al., 2018). Thus, INF1 was used as positive control. In VIGS assay, 3 weeks after VIGS-mediated gene silencing, *N. benthamiana* leaves were agroinfiltrated with RP309, GFP, and INF1. As expected, INF1 induced cell death in TRV: GFP-treated control plants, but failed to induce cell death in BAK1- and SOBIR1-silenced plants. Similarly, RP309-triggered cell death in GFP-silenced plants but not in BAK1- and SOBIR1-silenced plants (Figure 7B). In addition, qRT-PCR analysis showed that the relative expression levels of BAK1 and SOBIR1 decreased by 70–80% in the corresponding silenced plants compared with the TRV: GFP treated plants, indicating that BAK1 and SOBIR1 genes had been effectively silenced (Figure 7C). Meanwhile, the protein expression was normal (Figure 7D). Collectively, RP309 as a potential HAMP molecule which activates the defense response and this response required co-receptors BAK1 and SOBIR1 in *N. benthamiana*.

### Functional Domain Prediction and Analysis of Homologous Genes in Other Organisms

**FIGURE 8:**
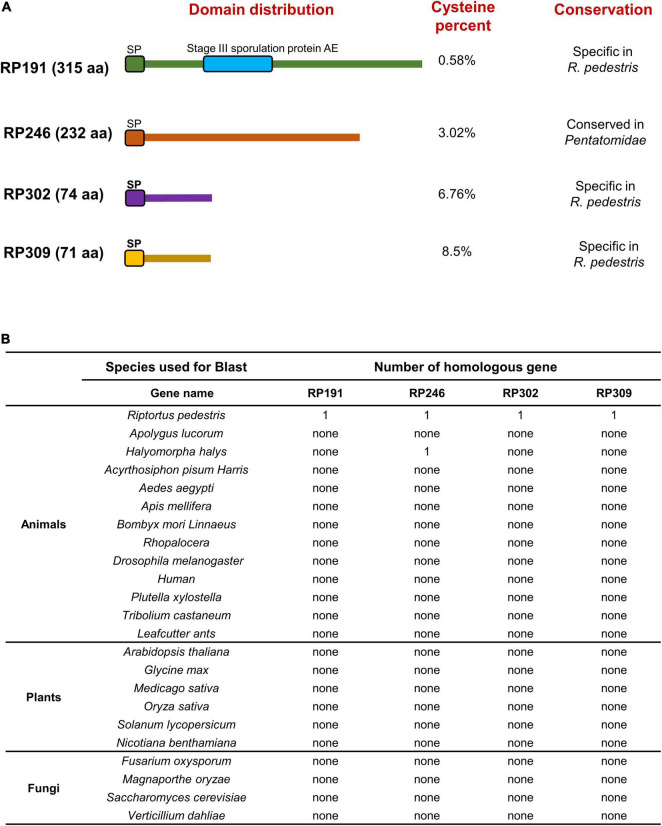
Functional domain prediction and analysis of homologous genes in other organisms. **(A)** Functional domain prediction of necrosis inducing proteins (NIPs). SP, signal peptide. **(B)** Homologous gene analysis of four NIPs using the Blast algorithm (NCBI) against other reported sequences.

We systematically analyzed the sequence features of the 4 candidate effectors and found that RP191 contains an additional stage III sporulation protein AE domain (PF09546), while the other 3 proteins have no characterized functional domains (Figure 8A). Some plant pathogen effectors contain cysteine-rich residues (Zhang et al., 2017; Wang et al., 2020), so we analyzed the cysteine content of the NIPs and found that only RP302 and RP309 were considered to be “cysteine rich” (>4%). We further investigated whether the 4 effectors are evolutionally conserved by performing a Blastp search against the NCBI non-redundant protein sequence database, including animals, plants, and fungi. Notably, no homologs of RP191, RP302, or RP309 were identified in other species, while homologous proteins of RP246 were found only in *Halyomorpha halys* and *Pristhesancus plagipennis*, within the family Pentatomidae. This indicates that RP191, RP302, and RP309 are unique to *R. pedestris*, while RP246 is conserved among the closely related Pentatomidae insects (Figure 8B).

## Discussion

Insect salivary proteins play an important role in the co-evolution of insects and plants, but there are few studies on the identification and function of *R. pedestris* salivary proteins. In this study, to explore the salivary secreted proteins of *R. pedestris* responsible for causing soybean cell death, the salivary glands were extracted and RNA-Seq was performed (Figures 1, 2). Three intracellular proteins, RP191, RP246, and RP302, and one apoplastic protein RP309 were found to induce tobacco cell death and ROS accumulation (Figure 3).

There has been a long-standing interest in functional analysis of plant pathogenic NIPs because their important roles in plant-pathogen interactions (Li et al., 2020). One major type of plant pathogen NIPs function in apoplastic spaces to activate plant immunity (Guo et al., 2019). RP302 are localized to the cytoplasm and nuclei, while RP246 and 191 are located in the ER. These NIPs are therefore probably not extracellular effectors, but rather intracellular effectors. A number of intracellular effectors that induce cell death promote plant pathogen colonization by dampening host immunity, including crinkling and necrosis proteins (CRNs) and RxLR (Wang and Wang, 2018; Huang G. Y. et al., 2019; He et al., 2020). Accordingly, RP191 and 246 promote plant pathogen infection, and RP302 induces *S. litura* feeding (Figure 5).

Herbivore-associated molecular pattern derived from saliva have been documented to activate an array of plant defenses against herbivores. Although RP309 can induce the most PTI marker genes expression and its function may depend on NbBAK1 and NbSOBIR1 (Figure 7), we need more evidence to prove that RP309 is a HAMP secreted by *R. pedestris*. For example, whether MAPK cascades can be activated and whether extracellular ROS can be induced. According to previous reports, most HAMP molecules are conserved (Zhou and Zhang, 2020; Snoeck et al., 2022), but RP309 is unique to *R. pedestris* (Figure 8). We suspect that RP309 is a specific reservation from the coevolution of *R. pedestris* and its host.

In conclusion, four candidate effectors, RP191, 246, 302, and 309, might be secreted into plant tissue during *R. pedestris* feeding, and can induce cell death in *N. benthamiana*. The expression of RP191 and RP246 in the ER, and RP302 in the cytoplasm and nuclei can inhibit plant immunity, thus promoting plant pathogen infection and insect feeding. These candidate NIPs are species-specific potential effectors that are only present in the Pentatomidae. We propose the following model to connect our findings with “staygreen syndrome.” During feeding, effectors, such as RP191, 246, and 302, are injected into plants to inhibit plant immunity and/or induce cell death, thus facilitating insect feeding. Consequently, the infested pods cease development, resulting in “staygreen syndrome,” because damaged seeds require less resource allocation from leaves. However, we cannot deny the functions of other candidate effectors that cannot induce cell death, and they may play a role in different signaling pathways, which is not clearly explained in this study, which is what we need to explore in the future. Overall, the present study provides insights into *R. pedestris* damage mechanisms, and the molecular functions of insect effectors, which may be useful for designing novel *R. pedestris* control strategies in the future.

## Data Availability Statement

The datasets presented in this study can be found in online repositories. The names of the repository/repositories and accession number(s) can be found below: NCBI Sequence Read Archive under accession numbers SRR18670484 (whole body) and SRR18670485 (salivary gland).

## Author Contributions

AX and DD conceived and designed the research. YD, XH, YY, JL, MZ, HS, YR, XL, and JT conducted the experiments. DS analyzed the data. AX and YD wrote the manuscript. All authors read and approved the manuscript.

## Conflict of Interest

The authors declare that the research was conducted in the absence of any commercial or financial relationships that could be construed as a potential conflict of interest.

## Publisher’s Note

All claims expressed in this article are solely those of the authors and do not necessarily represent those of their affiliated organizations, or those of the publisher, the editors and the reviewers. Any product that may be evaluated in this article, or claim that may be made by its manufacturer, is not guaranteed or endorsed by the publisher.
